# Mechanisms of Post-Replication DNA Repair

**DOI:** 10.3390/genes8020064

**Published:** 2017-02-08

**Authors:** Yanzhe Gao, Elizabeth Mutter-Rottmayer, Anastasia Zlatanou, Cyrus Vaziri, Yang Yang

**Affiliations:** 1 Department of Pathology and Laboratory Medicine, University of North Carolina at Chapel Hill, Chapel Hill, NC 27599, USA; evmutter@email.unc.edu (E.M.-R.); anastasia_zlatanou@med.unc.edu (A.Z.); cyrus_vaziri@med.unc.edu (C.V.); yang_yang1@med.unc.edu (Y.Y.); 2 Curriculum in Toxicology, University of North Carolina at Chapel Hill, Chapel Hill, NC 27599, USA

**Keywords:** DNA damage tolerance, post replication repair, DNA damage response, trans-lesion synthesis, template switching

## Abstract

Accurate DNA replication is crucial for cell survival and the maintenance of genome stability. Cells have developed mechanisms to cope with the frequent genotoxic injuries that arise from both endogenous and environmental sources. Lesions encountered during DNA replication are often tolerated by post-replication repair mechanisms that prevent replication fork collapse and avert the formation of DNA double strand breaks. There are two predominant post-replication repair pathways, trans-lesion synthesis (TLS) and template switching (TS). TLS is a DNA damage-tolerant and low-fidelity mode of DNA synthesis that utilizes specialized ‘Y-family’ DNA polymerases to replicate damaged templates. TS, however, is an error-free ‘DNA damage avoidance’ mode of DNA synthesis that uses a newly synthesized sister chromatid as a template in lieu of the damaged parent strand. Both TLS and TS pathways are tightly controlled signaling cascades that integrate DNA synthesis with the overall DNA damage response and are thus crucial for genome stability. This review will cover the current knowledge of the primary mediators of post-replication repair and how they are regulated in the cell.

## 1. Introduction

Accurate and efficient DNA replication is crucial for the health and survival of all living organisms. Under optimal conditions, the replicative DNA polymerases ε, δ, and α can work in concert to ensure that the genome is replicated efficiently with high accuracy in every cell cycle [1]. However, DNA is constantly challenged by exogenous and endogenous genotoxic threats, including solar ultraviolet (UV) radiation and reactive oxygen species (ROS) generated as a byproduct of cellular metabolism. Damaged DNA can act as a steric block to replicative polymerases, thereby leading to incomplete DNA replication or the formation of secondary DNA strand breaks at the sites of replication stalling. Incomplete DNA synthesis and DNA strand breaks are both potential sources of genomic instability [2]. As discussed elsewhere in this special issue, an arsenal of DNA repair mechanisms exists to repair various forms of damaged DNA and minimize genomic instability. Most DNA repair mechanisms require an intact DNA strand as template to fix the damaged strand. In this review, we will discuss the mechanisms behind Post-Replication Repair (PRR) that specifically help cells tolerate damage on the single stranded DNA template.

## 2. DNA Damage Repair and Complications at the Replication Fork

**Figure 1:**
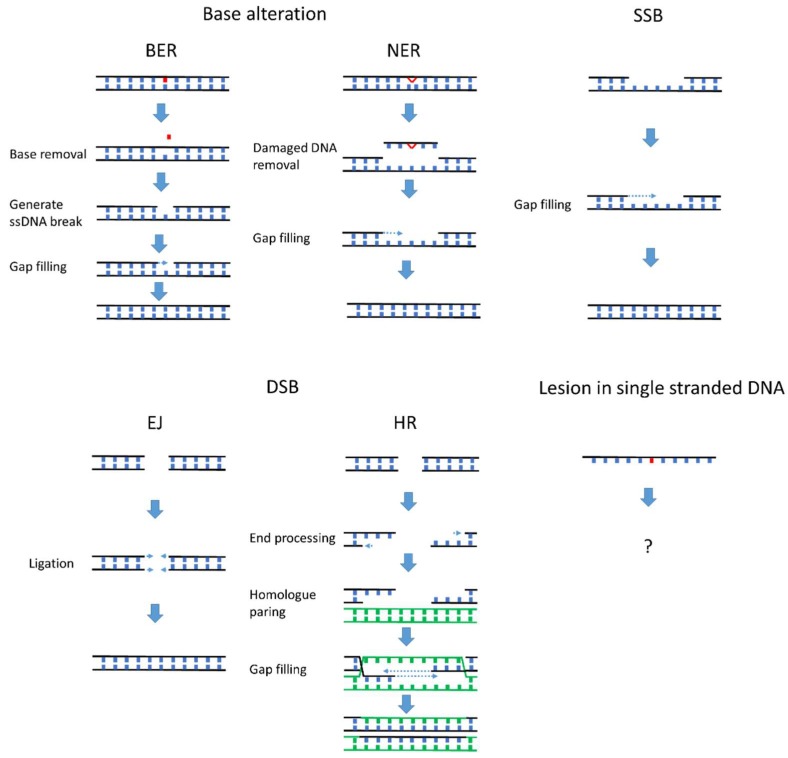
Many mechanisms efficiently repair DNA damage on the DNA double helix. Lesions in the double stranded DNA can be efficiently repaired by mechanisms corresponding to the specific type of DNA damage. Base-specific damage can be directly reversed by particular enzymes such as photolyases and O6-methylguanine DNA methyltransferase (MGMT) (reviewed in [4]). The majority of base-specific damage is repaired by base excision repair (BER) and nucleotide excision repair (NER). In BER and NER, the damaged base or surrounding DNA is excised from the double stranded DNA. The gap left behind is then filled by a DNA polymerase. Single stranded breaks (SSBs) are recognized by poly(ADP-ribose) polymerase 1 (PARP1), which activates downstream signaling that leads to gap-filling by DNA polymerases. Double stranded breaks (DSBs) are repaired by end joining (EJ) or by homologous recombination (HR). EJ directly ligates the exposed DSB with DNA ligase, while, during HR, break sites are replicated using undamaged homologous sequences of sister chromatid templates. In contrast, DNA lesions in single stranded DNA (ssDNA) cannot be repaired by BER, NER, HR, or EJ and must be remediated using alternative mechanisms (as suggested by the question mark in the figure). Post replication repair is a mechanism specialized in tolerating lesions in single stranded template.

DNA damage can be categorized by structural changes in the DNA such as base alteration, single stranded break (SSB), and double stranded break (DSB), each repaired via a distinct mechanism [3]. As summarized in Figure 1, a broad spectrum of DNA repair mechanisms has evolved to remove lesions that occur on double stranded DNA. Most DNA repair mechanisms rely on information from an undamaged DNA strand, either the complementary strand of the double helix (nucleotide excision repair (NER), base excision repair (BER) and SSB repair) or the sister chromatid and homologous allele (homologous recombination). Utilizing an undamaged template prevents aberrant alteration of the genetic coding on the damaged DNA strand. A major limitation to template-based repair mechanisms is that sometimes an undamaged DNA template strand is unavailable. This problem is frequently encountered during DNA replication, in the synthesis (S) phase of the cell cycle.

**Figure 2:**
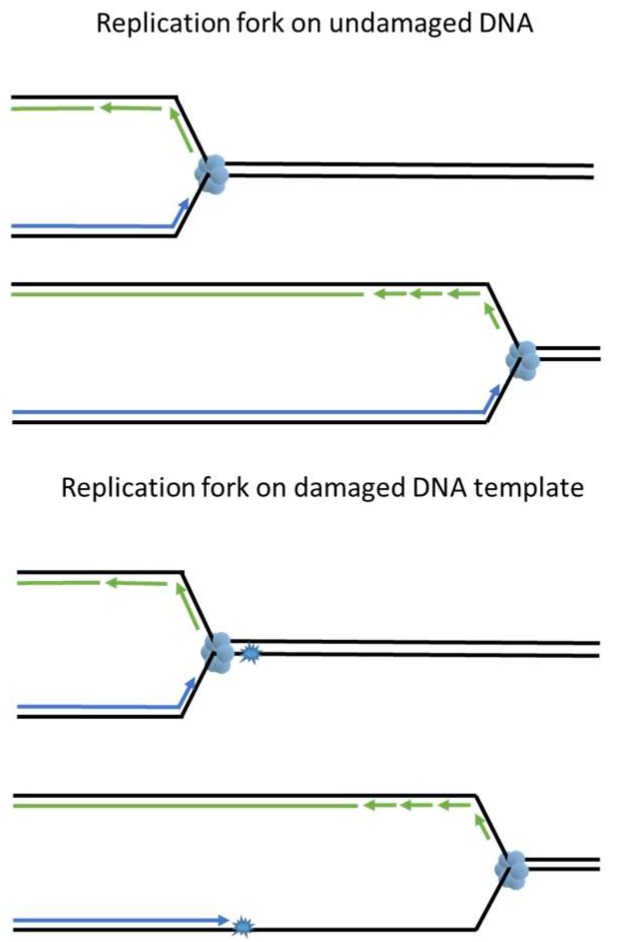
DNA lesions in single stranded DNA (ssDNA) are detrimental to the replication fork. DNA lesions on an ssDNA template act as road blocks for replicative polymerases but not for the replicative DNA helicase. An uncoupling of replicative DNA polymerase and DNA helicase activities generates single stranded DNA tracts. Persistent ssDNA is fragile and prone to breakage, generating lethal DSB.

DNA replication is a multistep process with two key events; (1) unwinding of the annealed double helix to expose ssDNA and (2) using this ssDNA as template to synthesize daughter strands. During an unperturbed S phase, DNA unwinding, carried out by the replicative helicase (the CDC45-MCM2-7-GINS or “CMG” complex), is strictly coupled with polymerase activity at replication forks (reviewed in [5]). Three replicative polymerases, pol ε on the leading strand and pol δ and pol α on the lagging strand, copy the template DNA with an error rate less than 10^−4^ [1]. The compact catalytic sites of replicative DNA polymerases confer high fidelity but preclude DNA damage-tolerant synthesis when using templates harboring bulky DNA lesions. As a result, replicative polymerases stall when a lesion is encountered (Figure 2). Fork-stalling DNA lesions are very prevalent in cells. In the human body there are approximately 30,000 lesions in every cell at any given time due to aerobic metabolism and endogenous depurination and deamination events [6]. It is inevitable that replication forks will be challenged by fork stalling lesions during DNA synthesis. Lesions encountered at replicating DNA are unique because the DNA in the vicinity of a replication fork is not double-helical. Excising the lesion from the ssDNA, as seen in BER and NER, will generate DNA strand breaks and result in fork collapse.

**Figure 3:**
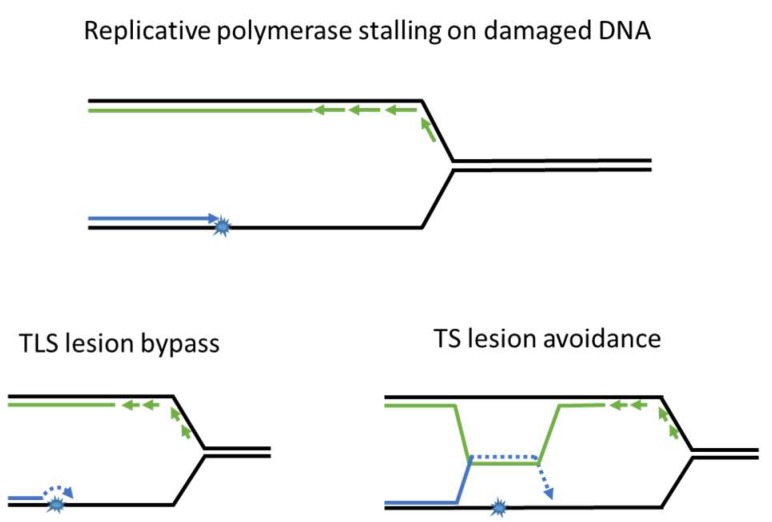
Post replication repair efficiently returns lesions in ssDNA to double helix. PRR utilizes trans-lesion synthesis (TLS) or template switching (TS) to bypass or avoid DNA lesions and prevent accumulation of ssDNA gaps. After being restored to its double-stranded state, damaged DNA may be repaired via the mechanisms described in Figure 1.

To survive fork-stalling DNA lesions, cells have developed post replication DNA repair mechanisms (PRR), which allow replication forks to progress through the lesions on damaged templates. The main role of PRR is to “patch” ssDNA gaps in the daughter strand and restore DNA to its double-stranded state for subsequent DNA repair via other mechanisms (covered in Figure 2 and Figure 3 and later sections of the paper). There are two mechanisms of PRR; trans-lesion synthesis (TLS), which employs TLS polymerases to directly replicate across the DNA lesion [7], and template switching (TS), which “borrows” the genetic information from the newly synthesized sister chromatid as a replication template [8] and thus avoids the lesion (Figure 3). As described in detail below, the TLS and TS pathways are coordinated to facilitate ongoing DNA synthesis on damaged genomes.

## 3. Activation of Post Replication Repair

Proliferating cell nuclear antigen (PCNA) is a ring shaped homo-trimeric protein complex that surrounds the DNA and is a central player in PRR. During the initiation of DNA replication, PCNA is loaded onto the chromatin by the Replication Factor C (RFC) clamp loader [9]. Upon completion of DNA replication, ATAD5 (Elg1 in yeast) unloads PCNA from chromatin [10,11]. Chromatin-bound PCNA slides along the DNA strand and serves as a processivity factor for DNA polymerases. In addition to tethering polymerases to template DNA, PCNA is a platform for a wide variety of proteins that participate in DNA replication and damage repair [12]. The interactions between PCNA and its binding partners are typically mediated by the PCNA inter-domain connecting loop (IDCL) and the PCNA interacting peptide (PIP) motif on its binding partner [13]. PCNA can also be modified by ubiquitin and SUMO (Small Ubiquitin Modifier) to create additional interfaces for binding partners during the S-phase or when the replication fork is under stress [12,14,15]. These post-translational modifications of PCNA are crucial events in PRR.

When the replication fork encounters a bulky DNA lesion, replicative polymerases stall but the MCM helicases continue unwinding the double helix ahead of the polymerase. The uncoupling of DNA polymerase and helicase at the stalled replication fork generates long stretches of ssDNA covered by replication protein A (RPA) that activates the DNA replication checkpoint [16]. RPA-coated ssDNA generated by fork-stalling recruits Rad18 (a PCNA-directed E3 ubiquitin ligase) to the vicinity of the DNA lesion [17,18,19]. Chromatin-bound Rad18 and its associated E2 ubiquitin-conjugating enzyme (Rad6) mono-ubiquitinate PCNA at the conserved residue, K164 [14,15]. Mono-ubiquitinated PCNA initiates PRR by recruiting TLS polymerases to replace the activity of replicative polymerase at the stalled replication fork [20,21,22]. Although RPA-coated ssDNA is necessary for Rad18 chromatin-binding, multiple regulators have been shown to modulate the recruitment of Rad18 to PCNA. For example, TLS Pol η can facilitate the Rad18-PCNA interaction by binding to both proteins with its C-terminus domain and enhancing PCNA ubiquitination [23]. NBS1 (mutated in Nijmegen Breakage Syndrome) interacts with Rad18 at the Rad6-interacting domain to help recruit Rad18 to damaged DNA [24]. Additionally, BRCA1 facilitates efficient recruitment of RPA and Rad18 to damaged DNA and promotes PRR [25]. SIVA1 physically bridges chromatin-bound Rad18 and its substrate PCNA and promotes PCNA ubiquitination. However, SIVA1 is not required for Rad18 recruitment to DNA damage sites [26]. Spartan/DVC1 interacts with Rad18 and PCNA and is necessary for UV-tolerance, although the molecular mechanism by which Spartan/DVC1 regulates PRR is unclear [27,28,29,30,31]. Of note, Rad18 might not be the only enzyme that mono-ubiquitinates PCNA. PCNA mono-ubiquitination has been observed in both Rad18^−/−^ DT40 cells and in Rad18 KO mice, suggesting other E3 ubiquitin ligases may use PCNA as a substrate [32,33,34].

PCNA K164 mono-ubiquitination can be further extended to K63-linked poly-ubiquitin chains by another E3 ubiquitin-ligase, which in yeast is Rad5 [14]. The interaction between Rad18 and Rad5 brings Ubc13/Mms2-Rad5 to the vicinity of stalled replication forks [35]. Ubc13/Mms2 and Rad5-mediated poly-ubiquitination of PCNA directs lesion avoidance using the TS pathway [36]. In *Xenopus laevis*, PCNA poly-ubiquitination is induced by DNA damage, although it is unclear why PCNA is also modified when replicating undamaged DNA in this system [37]. In humans, the two human Rad5 orthologues, SNF2 histone-linker PHD-finger RING-finger helicase (SHPRH) [38,39] and helicase-like transcription factor (HLTF) [40,41], mediate PCNA poly-ubiquitination. Although PCNA poly-ubiquitination is a less abundant modification than PCNA mono-ubiquitination [42], it is clear that the human RAD5 homologues do contribute to DNA damage tolerance [43]. There are also some studies suggesting that Rad5 has an Mms2-Ubc13-independent role in the TLS pathway [44,45,46].

SUMOylation of PCNA, catalyzed by UBC9 and Siz1/2, has also been observed on lysines K127 and/or K164 during normal DNA replication or following sub-lethal DNA-damaging treatments [14,15]. SUMOylated PCNA interacts with Srs2, a helicase that displaces the Rad51 recombinase from ssDNA. Since Rad51 is essential for DNA repair via homologous recombination, Rad51 displacement prevents recombinational repair [47,48]. Consequently, the inhibition of homologous recombination by Srs2 at the replication fork further limits the pathway choices to PRR when a DNA lesion is encountered on the single stranded template [49]. Interestingly, PCNA SUMOylation also has been shown to facilitate Rad18 E3 ligase activity towards PCNA by physically linking Rad18 and PCNA in yeast [50]. Although this is not an evolutionally conserved mechanism for Rad18 activation, it still exemplifies the cross talk between different PCNA modifications.

## 4. Trans-Lesion Synthesis

The Trans-Lesion Synthesis branch of PRR employs specialized DNA polymerases to perform replicative bypass of DNA lesions. In a process termed “polymerase switching” the TLS polymerases are recruited to stalled replication forks where they transiently replace the replicative polymerases. There are three TLS polymerases RAD30 (η), Rev1, and ζ, in budding yeast and two additional TLS polymerases, κ and ι, in vertebrates. Of these TLS polymerases, η, κ, ι, and Rev1 belong to the Y-family, while Pol ζ belongs to the B family [51]. The unique structure of TLS polymerases allows them to synthesize across lesions that block the conventional replicative polymerases. Compared to replicative polymerases, TLS polymerases have larger catalytic sites that are able to make loose contact with the template DNA and incoming nucleotide. This structure makes TLS polymerases more promiscuous in their selection of template DNA and allows them to accommodate templates with bulky adducts and abasic sites [52]. Furthermore, TLS polymerases lack the proofreading exonuclease domain that is present in the replicative ones and which is critical for accurate DNA synthesis. Therefore, utilizing TLS polymerases to replicate damaged templates can confer damage-tolerant DNA synthesis at the cost of reduced replication accuracy [7].

Although TLS polymerases are inherently error prone, TLS can be relatively error-free in instances when the “correct” Y-family polymerase(s) are recruited to bypass a cognate lesion. For example, in the presence of a UV radiation-induced thymine-thymine cis-syn cyclobutane dimer (CPD), DNA Pol η preferentially incorporates two adenines (A) opposite the thymine-thymine dimer to accurately bypass the lesion [53]. Similarly, Pol κ accurately bypasses bulky (BP)-7,8-diol-9,10-epoxide-N(2)-deoxyguanosine (BPDE-dG) adducts induced by the environmental carcinogen Benzo[a]pyrene [54,55]. Moreover, Pol ι frequently incorporates a correct base (cytosine, C) following the oxidative lesion 8-oxoguanine as well as 2-Acetylaminofluorene (AAF) adducted guanine [56]. Thus TLS polymerases are capable of contributing to DNA damage tolerance and S-phase progression without compromising genome stability.

Due to the intrinsic ability of TLS polymerases to accommodate a wide variety of lesions, sometimes lesion bypass can be carried out by a “non-ideal” error-prone polymerase, especially when the “correct” polymerase is not available. This phenomenon of compensatory error-prone lesion bypass by inappropriate DNA polymerases is exemplified by xeroderma pigmentosum variant (XP-V) patients, in which Pol η is mutated [57]. XP-V patients experience extreme sunlight sensitivity and have an increased incidence of skin cancer. In XP-V patients, UV-induced DNA damage is bypassed by other Y-family DNA polymerases such as Pol ι [58] and Pol κ [59], resulting in high mutation rates. These studies suggest that, despite the presence of intact nucleotide excision repair, selecting the correct TLS polymerase to accurately bypass the DNA lesion is crucial for the prevention of elevated mutagenesis.

**Figure 4:**
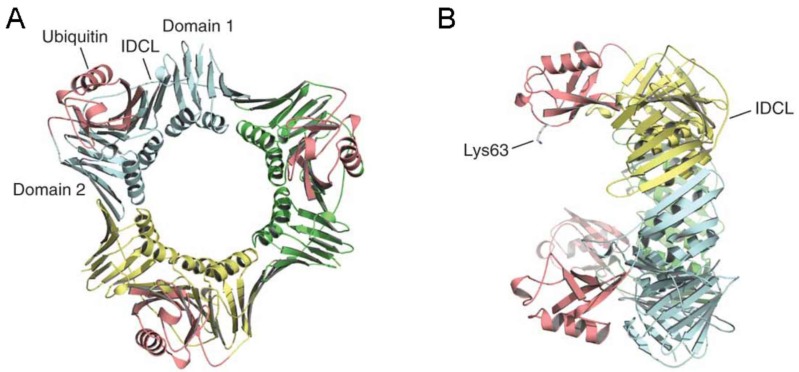
Structure of a monoubiquitinated PCNA ring (picture from reference [22]). (**A**) Back view of monoubiquitinated PCNA ring showing the two domains of a single PCNA subunit and the inter-domain connecting loop (IDCL). Ubiquitin is shown in red. Three individual PCNA molecules (shown in blue, green, and yellow) constitute the ring shape; (**B**) Side view of the monoubiquitinated PCNA where the back surface is to the left and the front surface is to the right of the figure. Notice that the ubiquitin is located on the back surface of the PCNA ring while IDCL is to the front side of the PCNA ring.

As mentioned previously, the recruitment of TLS polymerases to stalled replication forks is facilitated by Rad18-mediated PCNA mono-ubiquitination. TLS polymerases possess a higher affinity towards PCNA in its mono-ubiquinated state [20,21] and may displace the processive DNA polymerases to replicate through damaged DNA [60]. Interestingly, lysine 164 of PCNA is not located at the IDCL, the protein-protein interacting domain on PCNA that mediates the interaction with the PIP motif of target proteins [13]. Instead, the K164-linked ubiquitin is attached to the back face of PCNA, creating a distinct interacting motif for TLS polymerases [22] (Figure 4). In addition to a PIP-motif, all Y family polymerases contain at least one Ubiquitin-Binding Zinc finger (UBZ) or Ubiquitin-Binding Motif (UBM) at the C-terminus of the protein [61]. The ubiquitin-binding domain, together with the PIP motif on TLS polymerases, mediates the preferential interaction with mono-ubiquitinated PCNA. In this structure, the binding of Pol η to PCNA does not interfere with the binding of Pol δ. Instead, Pol η is resting at the back face of PCNA, while Pol δ is contacting the front surface of PCNA [22] (Figure 4). It is important to note that PCNA is a trimeric ring, which in theory could interact with three DNA polymerases at the same time. In fact, the structural study by Freudenthal et al. favors the notion that the PCNA ring acts as a molecular ‘tool belt’, carrying both TLS and replicative polymerases to cope with damage on ssDNA, similar to the β sliding clamp in E.coli [62]. Unlike the Y-family TLS polymerases (η, κ, ι, REV1), Pol ζ (a B-family DNA polymerase) does not contain a UBZ domain. However, Pol ζ recruitment to stalled replication forks is mediated by the Y family polymerases, such as REV1 [63], and therefore might have some dependency on PCNA mono-ubiquitination.

Despite the extensive studies suggesting that PCNA and its ubiquitination facilitate TLS activation, the absolute requirement of PCNA mono-ubiquitination is still being debated. Several lines of evidence suggest that TLS can, in some instances, proceed without the need for PCNA mono-ubiquitination. Gueranger and collegues showed that the Pol η PIP-box mutant could completely restore the UV resistance in a pol η-deficient cell line [64]. Acharya and colleagues additionally found that the ubiquitin-binding domain of pol η is dispensable for its TLS function [65,66]. Embryonic fibroblasts from a genetically-engineered PCNA K164R “knock-in” mouse show some attenuation of TLS activity, yet retain lesion bypass activity [67]. In a recent study, pol η was shown to interact with unmodified and mono-ubiquitinated PCNA with equivalent affinities in vitro. Furthermore, mono-ubiquitinated PCNA did not enhance the lesion bypass activity of pol η [68]. Together, these studies suggest that PCNA ubiquitination is important for high-capacity TLS and efficient recruitment of TLS polymerases in normal cells. However, TLS may also occur in the absence of PCNA mono-ubiquitination, most likely due to residual UBZ-independent interactions between PCNA and the PIP motifs.

Even with our mechanistic understanding of trans-lesion synthesis, it is still not known how cells recruit appropriate and specific TLS polymerases to their cognate DNA lesions. It is formally possible that unknown factors “read” the structure of distorted DNA and then signal for the recruitment of the specific polymerases. However, as clearly documented in XP-V cells, CPD lesions can be bypassed by non-cognate TLS polymerases when Pol η is absent. Therefore, it is possible that mono-ubiquitinated PCNA does not discriminate between different TLS polymerases and serves as a recruitment platform that interacts with all TLS polymerases equally. Perhaps all the TLS polymerases are recruited to the damage site randomly and attempt to replicate through the lesion. In this “trial-and-error” mechanism, the “correct” polymerase bypasses a cognate lesion with the lowest energy expenditure. Non-ideal TLS polymerases would only perform a bypass when the “correct” polymerase is unavailable. Therefore, it might be the nature of the lesion itself that determines which polymerase engages and replicates across a specific type of DNA damage.

## 5. Template Switching

The Template Switching branch of PRR enables the stalled replication fork to use the newly synthesized daughter strand as template to avoid damaged DNA (Figure 3). Similar to TLS, TS is also mediated by PCNA post-translational modifications, specifically poly-ubiquitination and SUMOylation. The extension of Rad18-induced K164 mono-ubiquitination to poly-ubiquitination by Ubc13-Mms2 and Rad5 redirects the PRR mode to TS.

Most of our understanding of TS was generated from a series of elegant studies in yeast, which provided the basis for the existence of an error-free form of PRR [69,70]. This error-free mechanism requires Rad5 [71,72], Ubc13/Mms2 [73,74], DNA Pol δ [75], a subset of the RAD52 epistasis group [76] and involves recombination between partially replicated sister strands [77]. A groundbreaking study by Branzei and colleagues combining 2D gel electrophoresis of DNA replication intermediates and yeast genetics identified TS intermediates and defined their relationship with the previously mentioned TS factors [78].

Template switching, involves the formation of an X-shaped recombination intermediate like structure consisting of sister chromatic junctions (SCJs) close to the stalled replication fork. SCJ formation requires Rad51 and the resolution of SCJs structures depends on the Sgs1 helicase [78]. The SCJs generated during TS resemble the properties of a DNA crossover intermediate in homologous recombination [79]. A recent study visualized the recombination intermediates using electron microscopy and proved that the undamaged sister chromatid is used as template using a recombination-based mechanism [80]. During TS, the ssDNA template containing the DNA lesion anneals with the newly synthesized double stranded sister chromatid to form a three-strand duplex. This intermediate then releases the newly synthesized, undamaged daughter strand from the parental strand so that it can be used as template for damage avoidance. The structures formed in this process are later resolved by the Sgs1-Top3-Rim1 complex [80]. In addition to the core TS participants such as Rad5, Rad51, and Sgs1, there is a growing body of evidence that other DNA replication and damage repair factors are also involved in TS. For example, the 9-1-1 complex and Exo1 nuclease are essential for the initiation of TS [81], and Ctf4 was found to establish the connection between Pol α/primase and the MCM helicase to protect the replication fork structure that favors TS [82].

Although Ubc13-Mms2 and Rad5-mediated poly-ubiquitination of PCNA at K164 is a crucial event in TS, the structure and function of the PCNA poly-ubiquitin chain formed during TS remains elusive. In contrast, the significance of PCNA SUMOylation has been studied in more detail and is better understood. PCNA SUMOylation occurs both during normal, unperturbed DNA replication and in response to DNA damage [14,15]. SUMOylated PCNA provides an interaction platform for the recruitment of the Srs2 helicase [47,48]. Similar to other PCNA-interacting proteins, Srs2 contains a non-canonical PIP box motif that mediates PCNA binding. However, the interaction between the Srs2 PIP box and PCNA is fairly weak until a second interaction is established between SUMOylated PCNA and a SUMO-interacting motif at the C terminus of Srs2 [83]. PCNA-bound Srs2 helicase functions as a safeguard that limits unscheduled recombination at the replication fork by disrupting Rad51 filament formation on ssDNA during normal replication [84,85]. A small controversy still exists regarding why PCNA SUMOylation is required for the TS pathway; Srs2 actively removes Rad51 from the replication fork while template switching requires Rad51 activity. For this reason, it is generally believed that SUMOylation antagonizes the effect of PCNA ubiquitination and inhibits the TS pathway [14,15].

A recent study may help resolve this paradox; a SUMO-like domain protein, Esc2, was found to be recruited to stalled replication forks and displace Srs2, thereby creating a microenvironment that is permissive for Rad51 chromatin-binding [86]. Therefore, PCNA SUMOylation facilitates the usage of PRR on a challenged replication fork by suppressing homologous recombination. When TS is initiated by PCNA poly-ubiquitination, replication fork binding factors such as Esc2 alleviate the inhibition of recombination by PCNA SUMOylation and allow DNA damage avoidance [47].

Error-free DNA damage avoidance is a conserved PRR mechanism in metazoans [43,87]. Although the identity of the human Srs2 orthologue is still being debated, human PCNA SUMOylation has also been shown to suppress unscheduled DNA recombination via PARI (PCNA-associated recombination inhibitor), suggesting a conserved mechanism of regulating HR at the replication fork [88,89,90]. PCNA is also poly-ubiquitinated in human cells in response to DNA damage. Blocking K63 linked poly-ubiquitination chain formation sensitizes cells to DNA damage, increases UV-induced mutagenesis, and increases the reliance of cells on TLS for DNA damage tolerance [43]. Rad5 has evolved into two orthologues, SHPRH [38,39] and HLTF, in higher organisms [40,41]. Both SHPRH and HLTF can poly-ubiquitinate PCNA in vitro but via distinct mechanisms. SHPRH extends Rad18 mediated PCNA mono-ubiquitination, while HLTF transfers the pre-assembled poly-ubiquitin chain to Rad6-Rad18 and eventually onto unmodified PCNA [38,39,91]. Depletion of SHPRH and HLTF sensitizes the cell to DNA damaging agents and reduces PCNA poly-ubiquitination; however, *SHPRH^−/−^HLTF^−/−^* double knockout mouse embryonic fibroblasts are still able to poly-ubiquitinate PCNA, suggesting that other Rad5 orthologues might exist in higher organisms [92]. In addition to poly-ubiquitination of PCNA, HLTF has acquired additional functions in DNA damage tolerance. In response to UV damage, HLTF is able to mono-ubiquitinate PCNA and promote Pol η recruitment [93]. Furthermore, HLTF can also facilitate DNA strand invasion and D-loop formation in a Rad51-independent manner [94].

In addition to TS, there are other recombination-based mechanisms, such as complementary strand transfer repair (CSTR) [95] and replication fork reversal [96,97,98,99], that have also been shown to contribute to DNA damage avoidance.

## 6. Timing of Post Replication Repair

Both major modes of PRR are used to cope with collisions between DNA polymerases and lesions on the single-stranded DNA template. For this reason, PRR is critically important during S-phase of the cell cycle, when the DNA duplex is unwound and vulnerable to injury. In fact, cells have developed sophisticated mechanisms to control the timing of DNA post replication repair by limiting the availability of crucial PRR factors [100,101,102]. Interestingly, two studies using temporally controlled expression of Rad18 or Pol η found that it is possible to delay the onset of PRR without significantly affecting cell viability. Moreover, limiting PRR in the G2/M phase of the cell cycle does not significantly delay the progression of the S-phase [103,104]. These studies suggest that it is possible to detach the PRR with bulk DNA synthesis in the S-phase without compromising its function.

Nevertheless, the delayed onset of PRR during S-phase could potentially lead to the accumulation of dangerously long and fragile ssDNA stretches, especially on the leading strand. Exposed ssDNA in cells is frequently observed when the replicative polymerase is blocked. However, these ssDNA gaps are usually small in size and are located inside a single replicon, regardless of whether they are on the leading or the lagging strand. However extremely long ssDNA gaps (>3 kb) are rarely observed. This suggests that the leading strand is also synthesized discontinuously when replicating a damaged DNA template, similar to the discontinuous synthesis of the lagging strand [105].

Restart of replication requires a de-novo re-priming mechanism downstream (3′) of the stalled leading strand DNA polymerase. This repriming activity is carried out by DnaG in *E. coli* [106], and by a specialized polymerase PrimPol in higher organisms [107,108,109,110]. This repriming mechanism of PRR explains why UV-induced lesions only cause a slight reduction in fork speed even when Pol η is mutated in human cells [111]. The ability of PRR to function distal (5′) to a newly-primed leading strand may provide ample time to select the optimal DNA damage tolerance mechanism. It is also well established that TLS is functional outside the S phase of the cell cycle and can patch ssDNA arising in the G0 and G1 phases [112,113,114].

## 7. Conclusions and Outlook

Although neither TLS nor TS directly repair DNA damage, both PRR mechanisms enable an immediate response to polymerase stalling DNA lesions. PRR during S phase prevents gross chromosomal rearrangements and ensures that replication is completed in a timely manner.

A deficiency in PRR could lead to replication fork collapse and the accumulation of DNA DSBs. In the absence of PRR, DSB repair mechanisms could allow for tolerance of replication-associated DNA damage. However, DSB repair pathways have limitations; DNA end-joining frequently results in mutations, while HR serves as the salvage pathway and creates complex and unstable repair intermediates through the use of a homologous strand from another DNA molecule. (For more insight into salvage and other homologous recombination-mediated DNA damage tolerance, we invite readers to read a recent review on this topic [115]) Therefore, PRR is perhaps the least genome-destabilizing option for the tolerance of DNA lesions arising in S-phase.

Both branches of PRR are important for cells to tolerate and survive DNA damage. In terms of maintaining genome stability, TS has a great advantage over TLS because it does not induce base mutations. Because of its intrinsic error-propensity, TLS has been linked to both increased mutation rates and might, therefore, fuel carcinogenesis [116,117,118]. In established cancers, TLS is also suggested to be responsible for a high mutation frequency and elevated treatment resistance [116,119,120]. However, it is not known why untransformed cells would utilize the error-prone TLS pathway when error-free TS is available.

Many DNA damaging chemotherapy agents cause lesions that can be tolerated by PRR. Such lesions include bulky adducts generated by alkylating agents and the DNA crosslinks produced by platinating agents. For this reason, cancer cells could upregulate PRR to survive therapy-induced DNA damage. Interestingly, a recent report identified a cancer cell-specific mechanism of TLS activation that might provide a general paradigm for how tumors acquire both mutability and DNA damage tolerance via pathological PRR [121]. Therefore, targeting PRR pathways individually, or in combination with compensatory genome maintenance mechanisms, could sensitize cancer cells to intrinsic and therapy-induced replicative stress.
